# GPR21 Inhibition Increases Glucose-Uptake in HepG2 Cells

**DOI:** 10.3390/ijms221910784

**Published:** 2021-10-05

**Authors:** Gemma K. Kinsella, Stefania Cannito, Valentina Bordano, John C. Stephens, Arianna C. Rosa, Gianluca Miglio, Valeria Guaschino, Valeria Iannaccone, John B. C. Findlay, Elisa Benetti

**Affiliations:** 1 School of Food Sciences and Environmental Health, Technological University Dublin, Grangegorman, D07 ADY7 Dublin, Ireland; gemma.kinsella@tudublin.ie; 2 Department of Clinical and Biological Sciences, University of Turin, 10125 Turin, Italy; stefania.cannito@unito.it; 3 Dipartimento di Scienza e Tecnologia del Farmaco, University of Turin, Via Pietro Giuria 9, 10125 Turin, Italy; valentina.bordano@unito.it (V.B.); ariannacarolina.rosa@unito.it (A.C.R.); gianluca.miglio@unito.it (G.M.); valeria.guaschino@edu.unito.it (V.G.); valeria.iannaccon@edu.unito.it (V.I.); 4 Department of Chemistry, Maynooth University, Maynooth, Co. Kildare, Ireland; john.stephens@mu.ie; 5 Kathleen Lonsdale Institute for Human Health Research, Maynooth University, Maynooth, Co. Kildare, Ireland; 6 Department of Biology, Maynooth University, Maynooth, Co. Kildare, Ireland; j.b.c.findlay@leeds.ac.uk; 7 School of Biomedical Sciences, University of Leeds, LS2 9JT Leeds, UK

**Keywords:** GPR21, GPCRs, hepatocytes, hepatic insulin resistance

## Abstract

GPR21 is a constitutively active, orphan, G-protein-coupled receptor, with in vivo studies suggesting its involvement in the modulation of insulin sensitivity. However, its precise contribution is not fully understood. As the liver is both a major target of insulin signalling and critically involved in glucose metabolism, the aim of this study was to examine the role of GPR21 in the regulation of glucose uptake and production in human hepatocytes. In particular, HepG2 cells, which express GPR21, were adopted as cellular models. Compared with untreated cells, a significant increase in glucose uptake was measured in cells treated with siRNA to downregulate GPR21 expression or with the GPR21-inverse agonist, GRA2. Consistently, a significantly higher membrane translocation of GLUT-2 was measured under these conditions. These effects were accompanied by an increased ratio of phAKT^(Ser473)^/tot-AKT and phGSK-3β^(Ser9)^/tot-GSK-3β, thus indicating a marked activation of the insulin signalling pathway. Moreover, a significant reduction in ERK activation was observed with GPR21 inhibition. Collectively, these results indicate that GPR21 mediates the negative effects on glucose uptake by the liver cells. In addition, they suggest that the pharmacological inhibition of GPR21 could be a novel strategy to improve glucose homeostasis and counteract hepatic insulin resistance.

## 1. Introduction

Type 2 diabetes (T2D) mellitus is a chronic disease that is reaching epidemic proportions, and despite several therapeutic options being available, almost half of patients do not achieve their treatment goals [1]. Insulin resistance plays a crucial role in its pathogenesis and, interestingly, represents the best predictor of a future diagnosis of this pathology [2]. Generally, T2D is preceded by impaired glucose tolerance, characterised by abnormally increased postprandial blood glucose levels due to impaired insulin sensitivity. Currently, it has been estimated that 347 million people have an impaired glucose tolerance [3], which is itself associated with increased cardiovascular mortality [4]. On this basis, advancing knowledge of the molecular mechanism underlying insulin sensitivity impairment is necessary to identify novel targets for potential pharmacological strategies to counteract its establishment and progression.

G-Protein Coupled Receptors (GPCRs) are a superfamily of transmembrane receptors that perform a wide range of biological functions within the human body [5,6,7]. They represent a rich source of drug targets: approximately 30% of marketed drugs act through these receptors [8]. However, orphan receptors still comprise ~25% of the targetable GPCR space and are attracting particular interest in the drug discovery field as they may represent novel therapeutic targets for a range of conditions [9].

The Rhodopsin subfamily is the largest subset grouping of GPCRs with diverse ligands including neurotransmitters, hormones, and lipids. GPR21 is a broadly expressed, orphan, rhodopsin-like receptor that shows constitutive activity through Gαq type G proteins, in particular Gα q and Gα 15/16 [10,11]. Interestingly, this receptor has been shown to be involved in the pathogenesis of insulin resistance, thus representing a potential new target for the treatment of Type 2 diabetes [11,12].

**Figure 1:**
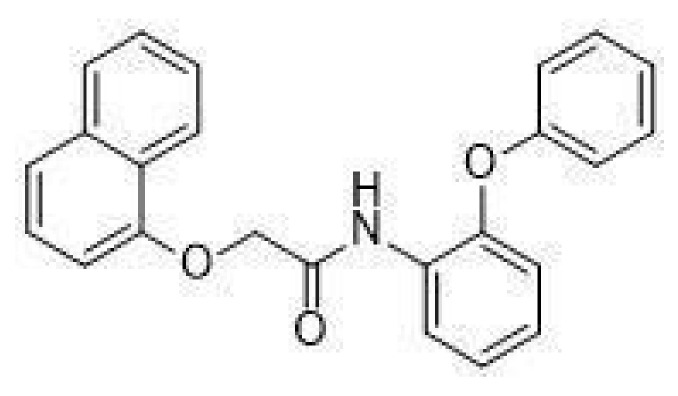
Structure of GRA2, (2-(1-naphthyloxy)-N-(2-phenoxyphenyl)acetamide.

In particular, in vivo studies on GPR21 knockout (KO) mice demonstrated that the deletion of this receptor improves glucose tolerance and systemic insulin sensitivity in animals fed with a high-fat diet [13,14]. The mechanism by which GPR21 exerts its metabolic phenotype is difficult to pinpoint, with Osborn et al. suggesting that GPR21 may be a novel control point coordinating macrophage pro-inflammatory activity in the context of obesity-induced insulin resistance, thus hypothesising an indirect role for this receptor in the induction of insulin resistance. Subsequently, we showed that GPR21 overexpression in HEK293T cells was associated with impaired insulin signalling, thus indicating a direct involvement in the establishment of insulin resistance [11]. In addition, we recently identified the acetamide GRA2, (2-(1-naphthyloxy)-N-(2-phenoxyphenyl)acetamide (Figure 1), which acts as an inverse agonist for this receptor and can counteract the influence of GPR21 on the insulin signalling pathway [11]. However, despite its intriguing potential, the effect of GPR21 in target cells for the action of insulin has not been investigated yet.

The aim of this study was to investigate the ability of this receptor to affect the insulin sensitivity of hepatocytes. As liver tissue is crucial for the regulation of glucose homeostasis, the development of insulin resistance in hepatocytes is expected to have important and serious systemic consequences [15]. To this purpose, the specific aims of this study were to investigate the effect of GPR21 in hepatocytes demonstrating its presence and activity and analysing its effect on glucose uptake/production and insulin signalling by (1) siRNA down-regulation or (2) its pharmacological inhibition by using the inverse agonist GRA2.

## 2. Results

### 2.1. Expression and Activity of GPR21 in HepG2 Cells

**Figure 2:**
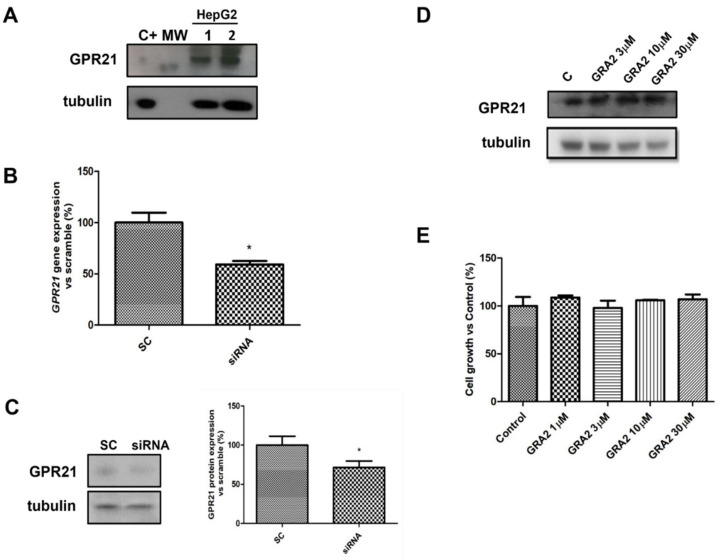
GPR21 receptor expression in HepG2 cells. (**A**). Representative Western blot analysis for the expression of GPR21 in HepG2 cells (MW: 37 kDa, C+: HT1080 whole cells lysate). Tubulin expression was assessed to confirm homogeneity of loading. (**B**). qPCR analysis for GPR21 mRNA in HepG2 cells transfected with non-silencing siRNA (SC) or with siRNA for GPR21 (siRNA). Data are expressed as mean ± SEM of three independent experiments run in triplicate. (**C**). Western blot analysis of GPR21 protein levels in HepG2 cells transfected with non-silencing siRNA (SC) or with siRNA for GPR21 (siRNA) (left panel). Equal loading was evaluated by re-probing the membrane with anti-tubulin. The relative densitometric analysis is reported in the right panel. (**D**). Western blot analysis of the GPR21 receptor exposed to either vehicle alone or GRA2 (3–30 µM, 24 h). Equal loading was evaluated by re-probing the membrane with anti-tubulin. (**E**). MTT assay on HepG2 cells exposed to either vehicle alone or increasing concentrations of GRA2 (1–30 µM) for 24 h. Cell growth was expressed as a percentage of the control cultures (100%). Data are expressed as mean ± SEM of three independent experiments run in triplicate. * *p* < 0.05 vs. scramble control (SC).

To evaluate whether HepG2 cells were suitable cellular models to study the role of GPR21 in hepatocytes, the expression of this receptor was first assessed using a Western blot analysis. As shown in Figure 2A, GPR21 was detected in HepG2 cells. As GPR21 is a constitutively active receptor [10], the effects of its inhibition were quantified. In particular, two strategies were adopted: a specific siRNA against GPR21 to decrease GPR21 expression (Figure 2B,C) and a GPR21-inverse agonist, GRA2 [11], to inhibit receptor activity. As shown in Figure 2B,C, GPR21 expression at the gene and protein levels was significantly reduced by using specific siRNA against GPR21. By contrast, cell exposure to GRA2 (3–30 µM, 24 h), did not affect GPR21 expression (Figure 2D). Moreover, GRA2 (1–30 µM, 24 h) did not affect cell viability as evaluated by the MTT assay (Figure 2E).

**Figure 3:**
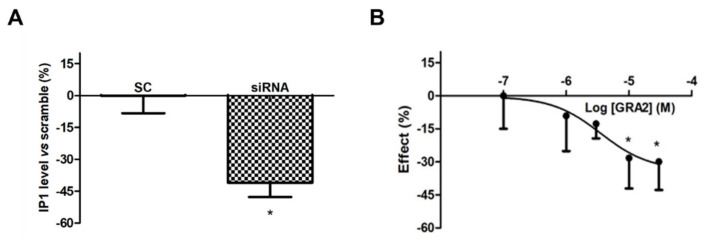
Effect of GPR21 gene silencing and GRA2 treatment on IP1 production. GPR21 constitutive activation was quantified by measuring the intracellular IP1 level in HepG2 cells transfected with non-silencing siRNA (SC, Scramble) or silenced with siRNA against GPR21 for 72 h (panel (**A**)) as well as in HepG2 cells treated with increasing concentrations of the inverse agonist (3–30 µM, for 1 h, panel (**B**)). Data are expressed as mean ± SEM of four independent experiments run in duplicate. Values are expressed in % vs. control or scramble. * *p* < 0.05 vs. control or scramble.

However, as shown in Figure 3A, the use of a specific siRNA against GPR21 significantly (*p* < 0.05) decreased IP1 levels, which appeared to be approximately 40% lower compared to the Scramble Control (SC)-treated samples. In addition, GRA2 decreased IP1 production levels in a concentration-dependent manner with a significant (*p* < 0.05) inhibition at concentrations above 10 µM (IC50 of 1.6 × 10^−6^ M, Figure 3B). These results suggest that GPR21 is a constitutively active receptor in HepG2 cells and that GRA2 was able to act as an inverse agonist in our experimental model. Therefore, as indicated by these results, HepG2 cells represent a suitable in vitro model to study the role of this receptor in hepatocytes.

### 2.2. Effect of GPR21 Gene Silencing and GRA2 Treatment on Glucose Uptake and Glucose Production in HepG2 Cells

**Figure 4:**
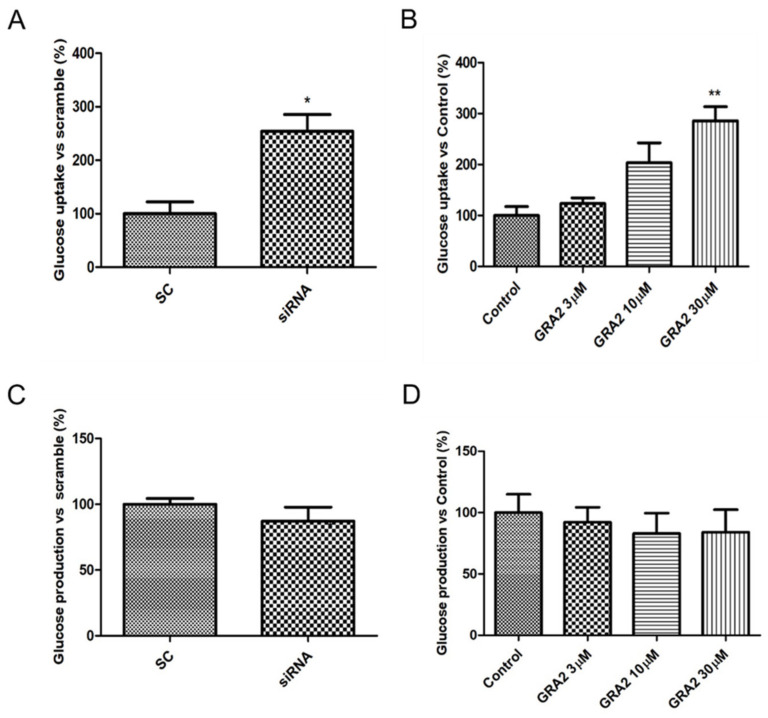
Effect of GPR21 gene silencing and GRA2 treatment on hepatic glucose homeostasis. Glucose uptake was assessed using a 2-NBDG fluorescent probe; HepG2 cells were transfected for 72 h with non-silencing siRNA (scramble, SC) or with specific siRNA against GPR21 (siRNA, panel (**A**)) or exposed to increasing concentrations of inverse agonist GRA (3–30 µM, 24 h, panel (**B**)). Data are expressed as mean ± SEM (*n* = 4) in % vs. control or scramble. * *p* < 0.05 vs. scramble control (SC); ** *p* < 0.01 vs. control. (**C**,**D**). Glucose production was evaluated on HepG2 cells transfected with non-silencing siRNA (SC) or with GPR21 siRNA (**C**) or exposed to increasing concentrations of inverse agonist GRA2 (3–30 µM, 24 h, panel (**D**)). Values are expressed in % vs. control or scramble. Data are expressed as mean ± SEM (*n* = 3) in % vs. control or scramble.

The liver plays a crucial role in glucose homeostasis, with glucose uptake by hepatocytes considered an essential element driving hepatic insulin resistance. To assess whether GPR21 contributes to the regulation of cellular glucose homeostasis, the effects of GPR21 gene silencing and of GRA2 on glucose uptake were measured. As shown in Figure 4A, GPR21 gene silencing significantly increased glucose uptake (*p* < 0.05) in comparison to HepG2 cells transfected with scramble control (SC) sequences. Consistently, in comparison to control cells, a concentration-dependent increase in glucose uptake was also measured in cells treated with GRA2 (Figure 4B). Additionally, we evaluated whether GPR21 affects glucose production in HepG2 cells. The inhibition of GPR21 by gene silencing (Figure 4C) or by GRA2 treatment (Figure 4D) did not affect cellular glucose production, suggesting that GPR21 could impair glucose homeostasis primarily through its effect on glucose uptake.

### 2.3. Effect of GPR21 Gene Silencing and GRA2 Treatment on GLUT-2 Expression

**Figure 5:**
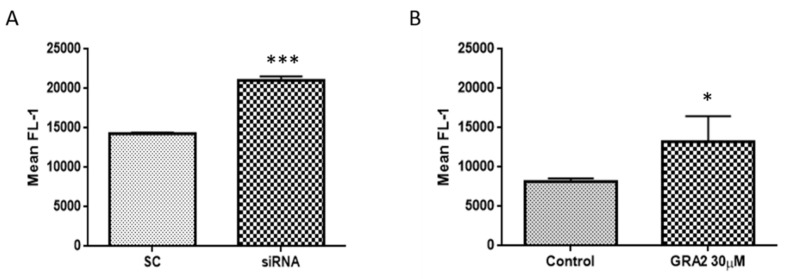
GPR21 inhibition improves GLUT-2 translocation to the plasma membrane. Flow cytometry analysis of GLUT-2 expression at the cell membrane of HepG2 cells transfected with non-silencing siRNA (SC) or with specific Scheme 21. (siRNA, (**A**)) or exposed to GRA2 (30 µM, 24 h, panel (**B**)). Data are expressed as the mean of fluorescence FL-1 ± SEM; *n* = 4. * *p* < 0.05 vs. control; *** *p* < 0.001 vs. scramble control (SC).

Since our results indicated an increased glucose uptake after the inhibition of GPR21, we investigated whether GPR21 can affect the membrane expression of the prominent glucose transporter mainly present in the liver, GLUT-2 [16]. We evaluated GLUT-2 expression in HepG2 cells by performing flow cytometry analysis (Figure 5 and Supplementary Figure S1). Our data showed that the selected silencing of GPR21 by specific siRNA (Figure 5A and Figure S1A) as well as the inhibition of GPR21 activity by GRA2 treatment (30 µM, Figure 5B and Figure S1B) resulted in the increased translocation of GLUT-2 to the HepG2 cell membrane, thus explaining and justifying the increase in glucose uptake.

### 2.4. Effect of GPR21 Inhibition on Insulin Signalling in HepG2 Cells

**Figure 6:**
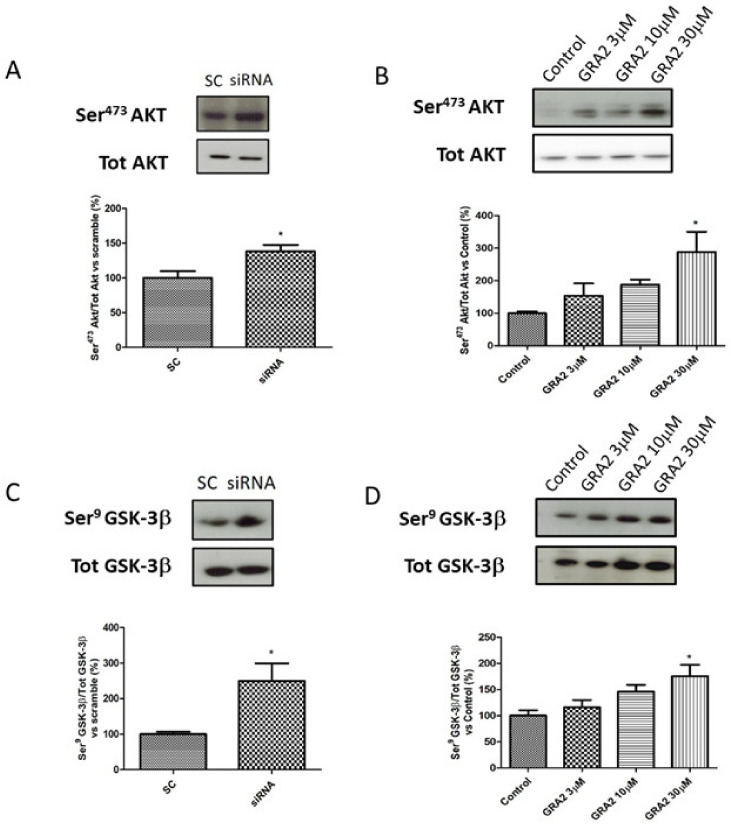
Effect of GPR21 inhibition on insulin signalling in HepG2 cells. Western blot analysis of phosphorylation levels of Ser^473^Akt and Ser^9^GSK-3β in HepG2 cells transfected for 72 h with non-silencing siRNA (scramble control, SC) or with specific siRNA against GPR21 (siRNA, panel (**A**,**C**)) as well as in HepG2 cells exposed to increasing concentrations of inverse agonist GRA2 (3–30 µM, 24 h, panel (**B**,**D**)). Equal loading was evaluated by a re-probing membrane with total Akt or GSK-3β. Densitometric analysis of the bands is expressed as relative optical density (O.D.) and was normalised using the related control band. Data are expressed as mean ± SEM; *n* = 4. * *p* < 0.05 vs. scramble control (SC) or control.

As AKT- GSK-3β signalling is a crucial regulator of GLUT-2 expression [17,18], which is affected by GPR21 inhibition, we evaluated the phosphorylation status of these target proteins. In particular, we evaluated the ratio of Ser^473^Akt/tot Akt and Ser^9^GSK-3β/tot GSK-3β. As shown in Figure 6, in HepG2 cells, gene silencing or the pharmacological inhibition of GPR21 induced an improvement of the insulin signalling pathway. In particular, our results demonstrated that the selected silencing of GPR21 receptors (by siRNA) resulted in a significant increase in the phosphorylation of Ser^473^Akt, which is essential for the full activation of this enzyme. Consistently, this effect was associated with a significant increase in the phosphorylation of Ser^9^ GSK-3β compared to SC (Figure 6A,C). GSK-3β is a constitutively active enzyme that could be inhibited by the phosphorylation of Ser^9^. Thus, an increase in the ratio of Ser^9^ GSK-3β/tot GSK-3β indicates an inhibition of its activity that results in an increased expression of GLUT-2. As shown in panel B, the same results were achieved in cells treated with GRA2. The effect was dose-dependent and became significant at the higher dose (*p* < 0.05, Figure 6B,D).

### 2.5. Effect of GPR21 Gene Silencing and GRA2 Treatment on ERK Activation

**Figure 7:**
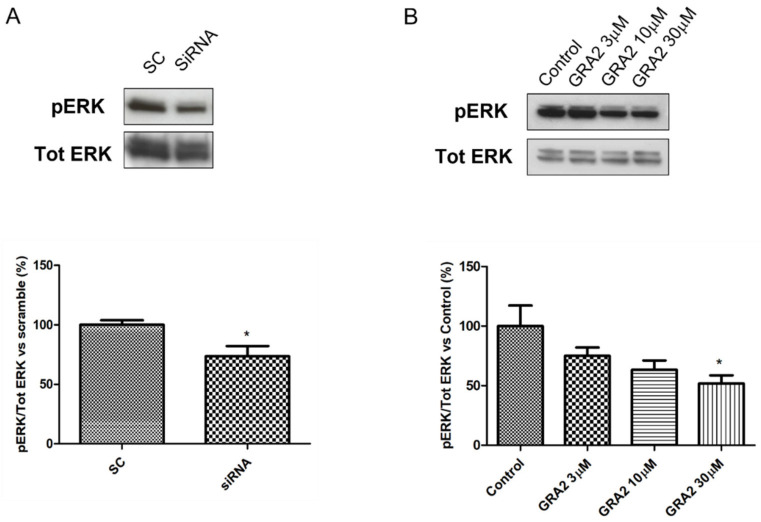
Effect of GPR21 gene silencing and GRA2 treatment on ERK activation. Western blot analysis of the phosphorylation levels of the MAPK ERK1/2 in HepG2 cells transfected for 72 h with non-silencing siRNA (scramble control, SC) or with specific Scheme 21. (siRNA, panel (**A**)) as well as in HepG2 cells exposed to increasing concentrations of the inverse agonist GRA2 (3–30 µM, 24 h, panel (**B**)). Equal loading was evaluated by a re-probing membrane with total ERK1/2. Densitometric analysis of the bands is expressed as relative optical density (O.D.) and normalised using the related control band. Data are expressed as mean ± SEM; *n* = 3. * *p* < 0.05 vs. scramble control (SC) or control.

As there is known cross talk between the insulin-AKT and MAPK/ERK signalling pathways [19] and that the insulin signalling could be negatively affected by ERK activation [20,21,22], we evaluated the effect of GPR21 inhibition on ERK phosphorylation. As shown in Figure 7, both gene silencing (Figure 7A) and the pharmacological inhibition of GPR21 (Figure 7B) induced a significant reduction in ERK phosphorylation, thus leading to a decrease in its activity. In particular, our results demonstrated that the inverse agonist GRA2 exerted a dose-dependent effect that became significant at the higher dose (*p* < 0.05).

## 3. Discussion

Insulin resistance is defined as the increased requirement for insulin to maintain glucose homeostasis and it is a consistent finding in patients affected by T2D [15]. T2D patients have elevated blood glucose levels due to an impaired pancreatic insulin production/secretion and a reduction in glucose uptake due to a decreased insulin activity in the peripheral organs, such as the liver and muscle. As the liver plays a central role in glucose metabolism by maintaining a balance between its uptake and storage, hepatic insulin resistance is thought to be largely responsible for the development of fasting hyperglycaemia [23]. While liver function is regulated by GPCRs, knowledge of how GPCRs regulate liver metabolism is limited [24]. A better understanding of the metabolic role of GPCRs in hepatocytes could lead to the development of novel drugs for the treatment of pathological conditions, including T2D.

This study focused on the orphan receptor, GPR21, which previous results have suggested is involved in the pathogenesis of insulin resistance [11,13,14], showing for the first time the presence of this receptor in HepG2 cells, and confirming its constitutive activity.

By inhibiting GPR21 with selected siRNA, we demonstrated that this receptor negatively affects glucose uptake and insulin signalling in hepatocytes. Interestingly, we also showed the possibility of counteracting GPR21 activity by using the inverse agonist GRA2.

Consistently, we observed a statistically significant increased expression of GLUT-2 on the membrane of the cells downregulated for GPR21 or treated with GRA2. GLUT-2 is the major glucose transporter expressed in hepatocytes [25]. The role of this high-capacity and low affinity glucose transporter is to take up glucose absorbed during feeding, preventing marked postprandial hyperglycaemia and to release it in the blood during fasting [16].

The expression of GLUT-2 is not correlated to hepatic glucose output. In vivo studies showed that the deletion of GLUT-2 suppressed glucose uptake but, unexpectedly, did not impair glucose output, thus suggesting that glucose trafficking across the membrane is differently mediated with respect to uptake and output and supporting the existence of a second pathway for glucose output [26,27,28]. These results are consistent with our data. We did not observe an increased glucose output in cells with the increased expression of GLUT-2.

However, the action of GLUT-2 is not limited to glucose transport. Several studies have indicated that it could be important for the control of glucose-sensitive gene expression in the liver. Hughes et al. suggested a role for GLUT-2 as a component of the glucose-sensing apparatus that regulates insulin release from the cells in response to changes in external glucose concentration [29]. More recently, Seyer et al. showed that hepatic GLUT-2 inactivation induces a long-term, progressive development of glucose intolerance, thus suggesting the existence of a liver/β cell axis that depends on normal liver glucose metabolism [30]. Interestingly, this suggests that GLUT-2 expressed by hepatocytes could have an important impact on β cell function, thus allowing us to speculate that GPR21 inhibition could also have an indirect but positive effect on beta cells.

In addition, GLUT-2 is markedly expressed in β-cells where it acts as a first messenger to trigger glucose signalling [18,31]. Interestingly, it has been observed that islet cells from human donors with Type 2 diabetes showed a significant (80–90%) reduction in GLUT-2 expression and lacked the glucose-stimulated insulin secretion (GSIS) response, a marker of beta cell dysfunction in Type 2 diabetes [32]. Consistently, a decreased GLUT2 gene expression was observed in the pancreatic cells of experimental models of diabetes and an impaired insulin secretion and synthesis was observed in KO mice for GLUT-2, but, interestingly, these effects were restored when the GLUT-2 was expressed again [33]. Our study is the first, to our knowledge, that has found a correlation between GPR21 activity and GLUT-2 expression, but on this basis, we can postulate that the effects of GPR21 inhibition deserve further investigation, in particular in the beta cells, where an increase in GLUT-2 could have an important impact. As several data have suggested that the AKT axis is crucial for the expression of GLUT-2 [18,34] and, in particular, that GSK3β is a negative modulator of its expression [17], we investigated the phosphorylation status of these two important protein targets of insulin signalling. Notably, we demonstrated that cells treated with GRA2 or siRNA for GPR21 showed an improvement of the insulin signalling pathways, as was evident by the observed increase in the Ser^473^Akt/tot Akt ratio. AKT phosphorylation of Ser^473^ is required to achieve full activation of this protein [35]. Strong evidence indicates that the Akt signalling pathway is key to mediating the effects of insulin on the anabolic metabolism in all organisms [36]. Studies on knockout animals for AKT showed that the absence of this enzyme in the liver induced severe insulin resistance, glucose intolerance, and a reduction in hepatic lipid synthesis, thus supporting the importance of its activation to improve insulin sensitivity [37,38]. One of the main targets of the action of AKT is GSK-3β, a constitutively active serine/threonine protein kinase enzyme that controls numerous cellular processes including glycogen metabolism [39]. GSK-3β is a peculiar enzyme that is inhibited, rather than activated, in response to insulin stimulation through the phosphorylation of Ser^9^ [40]. Consistent with the literature data, our results showed that GSK-3β inhibition is associated with an increase in GLUT-2 expression. In addition, since GSK-3β inhibits glycogen synthase by phosphorylation [40], the suppression of GSK-3β by AKT also leads to an increase in glycogen synthesis activity, thus allowing us to speculate that the genetic or pharmacological inhibition of GPR21 is associated with an increase in the synthesis of glycogen. However, this effect did not result in a reduction in glucose production. In fact, we did not observe significant differences in this parameter in the cells treated with selected GPR21 siRNA or GPR21 inverse agonist in comparison with the relative controls, thus suggesting that GPR21 seems not to affect gluconeogenesis.

In addition, our data showed that GPR21 inhibition decreased ERK phosphorylation. ERK belongs to the mitogen-activated protein kinase family and its activity is increased in several tissues of diabetic mice [41,42,43] and in primary cells from Type 2 diabetic patients [44]. Interestingly, it is recognised that the activation of this MAPKs negatively affects insulin signalling [20,22,45,46]. Consistently, the literature data has shown that the inhibition of the ERK pathway lowered the non-fasting blood glucose level, increased glucose tolerance, and improved insulin sensitivity in db/db mice and high-fat diet-fed KKAy mice [21,47]. On the other hand, it has been shown that mice deficient in the signalling adapter p62, an ERK inhibitor, had a high basal level of ERK activity and developed mature-onset obesity and insulin resistance [43,48]. On this basis we can speculate that the GPR21 deleterious effect could be, at least in part, mediated by ERK.

Overall, our results on GPR21 are supported by previous studies which have demonstrated that the selective stimulation of a Gq-linked GPCR expressed in hepatocytes leads to an impaired glucose tolerance [49]. Consistently, we showed that the inhibition of GPR21 activity significantly improved elements of the insulin signalling pathway, with an inhibition of GSK-3β and increased glucose cellular uptake. Our results are particularly relevant as they were achieved in a basal condition, thus confirming that being constitutively activated, GPR21 negatively affects insulin signalling. We can hypothesise that in several conditions, the activity of this receptor could increase over the controls, thus contributing to insulin signalling impairment in pathological conditions such as T2D. To this purpose, a recent paper by Romero-Nava et al. showed a change in the genetic expression of GPR21 in different in vivo experimental models of metabolic syndrome, thus suggesting its involvement in the pathogenesis of this condition and the hypothesis of a role for this receptor as a new therapeutic target [50].

This study has some limitations. First, as a fully in vitro study, this investigation was based on an experimental model of cell culture. Undoubtedly, primary cells are superior to permanent cell lines. However, the availability of human primary hepatocytes is very limited. We selected HepG2, which are cells that are frequently used to investigate hepatic signalling, because, despite their tumorigenic origin, they have been shown to be suitable to study insulin signalling [51]. Second, in our study, the effects observed with GPR21 gene downregulation were interestingly also evident after GRA2 treatment. However, the doses of the inverse agonist used in this study were quite high, in the µM range, thus suggesting that structure–activity relationship studies are necessary to optimise GRA2, which is the only GPR21 inverse agonist currently available, and achieve analogues with a higher potency.

Finally, we note that Wang et al. [52], by using a different methodology to achieve GPR21 KO mice, did not confirm the results previously achieved by Osborn and Gardner [13,14]. However, the experimental conditions were different, so it is not possible to arrive at a conclusive result without a direct comparison. In this context, our data are independent of, but consistent with, the results achieved by Osborn and Gardner and add useful information to better understand the role of this orphan receptor. Here, we demonstrated that GPR21 has a direct role on hepatic insulin sensitivity impairment, supporting previous results achieved in HEK293 cells [11].

In conclusion, we have shown that GPR21 negatively affects insulin sensitivity in hepatocytes, suggesting that its inhibition might represent a novel and promising pharmacological strategy to counteract the development of insulin resistance.

## 4. Materials and Methods

### 4.1. Cell Cultures

HepG2 cells (ATCC-HB-8065 from ATCC, USA) were cultured in Dulbecco’s modified Eagle’s medium-low glucose (DMEM, 1000 mg/L, Aurogene Srl, Rome, Italy) supplemented with L-glutamine (2 mM, Aurogene Srl, Rome, Italy), penicillin-streptomycin (100 µg/mL, Aurogene Srl, Rome, Italy) and foetal bovine serum (FBS, 10% *v*/*v*, Aurogene Srl, Rome, Italy), at 37 °C in a humidified 5% CO_2_ atmosphere incubator. HepG2 cells were seeded in normoxic conditions to obtain the desired sub-confluence level (65–70%) and to perform the experimental analyses.

### 4.2. Cell Viability Evaluation

Cells were plated (4 × 10^3^ cells/well) in 24-well culture plates and exposed to either the vehicle alone (DMSO, control) or GRA2. Cell viability was quantified in sub-confluent cultures by the 3-(4,5-dimethylthiazol-2-yl)-2,5-diphenyltetrazolium bromide (MTT, Sigma, Sigma-Aldrich, St Louis, MO, USA) colorimetric assay. The results were confirmed by determining cell density, as previously described [53,54].

### 4.3. Quantification of the Inositol 1-Phosphate (IP1) Level

Cellular IP1 levels were quantified by using an IP1 homogeneous time resolved fluorescence (HTRF) assay (Cisbio, PerkinElmer, Waltham MA, USA), as previously described [55,56]. Briefly, sub-confluent cell cultures were collected and re-suspended in the appropriate volume of the assay stimulation buffer. Cell suspensions were added to different concentrations of the compound to be tested and incubated at 37 °C for 1 h. Then, an IP1 lysis buffer containing IP-one-d2 conjugate was added to the appropriate wells, followed by the anti-IP-one cryptate Tb conjugate. Samples were incubated for 1 h at room temperature. The plate was read on a VICTOR X4 (PerkinElmer, Waltham, MA, USA) plate reader with emission at 615 nm and 665 nm. The fluorescence resonance energy transfer (FRET) ratio (665 nm/615 nm) was converted to IP1 concentrations by interpolating values from an IP1 standard curve.

### 4.4. siRNA-Mediated GPR21 Knockdown

RNA interference experiments to knockdown GPR21 expression in HepG2 cells were performed using Selected Negative Control siRNA (Silencer Select Negative control siRNA, Ambion, Thermo Fisher Scientific Inc., Rockford, IL, USA) or GPR21 siRNA (GPR21 Silencer Select Pre-designed siRNA cod. s6037, Ambion, Thermo Fisher Scientific Inc., Rockford, IL, USA) according to the manufacturer’s protocol.

HepG2 cells (1.2 × 10^6^ cells) were seeded and immediately transfected with a Transfection Reagent (Qiagen, Hilden, Germany) according to the manufacturer’s instructions up to 72 h. After 72 h, the HepG2 transfected cells were harvested for sample preparation.

### 4.5. RNA Isolation and Quantitative Real-Time PCR (qPCR)

GPR21 gene expression was quantified by a real-time qPCR. Total RNA was extracted by using TRI Reagent^®^ (Sigma, Sigma-Aldrich, St Louis, MO, USA) according to the manufacturer’s instructions. Complementary DNA synthesis and quantitative real-time PCR (q-PCR) reactions were performed on cell samples as previously described [57]. mRNA levels were measured by a q-PCR, using the SYBR^®^ green method as described [58] The amplification mix was prepared using iTaq Universal Syber Green SuperMix (Biorad Laboratories, Berkeley, CA, USA) following the manufacturer’s instructions and the real-time PCR was performed using Miniopticon ThermoCycler Instrument (Biorad Laboratories, Berkeley, CA, USA). A real-time amplification of human GPR21 was carried out using the following set of primers:

Human GPR21 FW 5′-TTTTCCACTGGGGCAAACCT-3′;

Human GPR21 RV 5′-TTGGCAGATGCGGAAGATGT-3′.

The housekeeping human gene glyceraldehyde-3-phosphate dehydrogenase (GAPDH) was amplified in parallel, in all amplification sets, using the following set of primers:

Human GAPDH FW 5′-TGGTATCGTGGAAGGACTCATGAC-3′;

Human GAPDH RV 5′-ATGCCAGTGAGCTTCCCGTTCAGC-3′.

### 4.6. Western Blot Analyses

About 30 µg of the total proteins were loaded for Western blot experiments, as previously described [53,59]. After blocking, the PVDF membranes were incubated at 4 °C overnight with antibodies against GPR21 (1 µg/uL, Abcam), p-Akt (1:1000, Cell Signaling Technology, #4051), Akt (1:1000, Cell Signaling Technology, #9272), p-Glycogen Synthase Kinase-3β (ph-GSK-3β, 1:1000, Cell Signaling Technology, #9322), GSK-3β (1:1000, Cell Signaling Technology, #9315), p-ERK (1:1000, Santa Cruz Biotechnology, sc-7383), ERK (1:1000, Santa Cruz Biotechnology, sc-94). To confirm equal protein loading, the membranes were stripped and incubated with an anti-tubulin (1:5000, Abcam) monoclonal antibody. Proteins of interest were detected with a horseradish peroxidase-conjugated secondary antibody (1:5000, Cell Signaling Technology) for 1 h at room temperature. The results were quantified using ImageJ software [60].

### 4.7. Glucose Uptake

HepG2 cells (1 × 10^4^ cells) were seeded in 96-well plates and after treatments, were incubated with 2-NBDG (Sigma, Sigma-Aldrich, St Louis, MO, USA) at a concentration of 40 μM for 1 h at 37 °C. Then, the cells were washed three times with cold PBS and the fluorescence intensity was immediately measured on the EnSight microplate reader (PerkinElmer, Waltham, MA, USA) at an excitation wavelength of 540 nm and an emission wavelength of 467 nm. An estimation of the overall glucose uptake was obtained by quantifying the fluorescence. The 4′,6-diamidino-2-phenylindole (DAPI) nuclear dye was used for normalization.

### 4.8. Glucose Production Assay

HepG2 cells (1.2 × 10^6^ cells) were seeded in 35 mm^2^ plates and after treatments they were washed three times to remove the remaining glucose. The medium was replaced with a glucose production buffer (glucose-free DMEM, without phenol red supplemented with 20 mM sodium lactate and 2 mM sodium pyruvate), as previously described [61]. After 3.5 h at 37 °C, the supernatants were collected to measure glucose concentration by the colorimetric assay Glucose Assay Kit MAK263 (Sigma, Sigma-Aldrich, St Louis, MO, USA) following the manufacturer’s instructions.

### 4.9. Flow Cytometry Analysis

HepG2 cells were seeded in 35 mm^2^ plates (1 × 10^6^ cells/dish) and cultured in the following experimental conditions: (i) untreated cells; (ii) HepG2 cells treated with insulin 100 nM for 1 h; (iii) HepG2 cells exposed to GRA2 30 µM for 24 h and (iv) HepG2 cells or transfected with non-silencing siRNA (SC) or with specific siRNA against GPR21 for 72 h. For each condition, the cells were rapidly washed with PBS, collected by trypsinization, and 5 × 10^5^ cells were resuspended in a staining buffer (PBS plus 1–2% of FBS). To the cellular suspension, human serum (20%) was added for 30 min at 4 °C and then GLUT-2 antibody (0.25 µg/106 cells) and Alexa Fluor 488 secondary antibody (1:500) for 30 min at 4 °C. After incubation with antibodies, 2 mL of a staining buffer was added to wash the excess of antibodies and then the cells were centrifuged at 1900 rpm for 5 min. The supernatants were discarded, and the cells were fixed with 400 µL of paraformaldehyde 1% (PAF) and analysed in flow cytometry. Detection of GLUT-2 green fluorescence (FL1) was performed on at least 5000 cells per sample with a FACScan equipped with a 488 nm argon laser using the CellQuest software (Becton-Dickinson, Milano, Italy). The peak of the FL1 intensity of GLUT-2-stained control cells was set to channel 101 and retained for all measurements.

### 4.10. Data Analysis

Data are presented as mean ± SEM. Statistical significance was evaluated by the Student’s t-test when applicable or a one-way analysis of variance (ANOVA) with a Bonferroni post-hoc test. The analyses were performed by using the GraphPad Prism version 5.0 for Windows (GraphPad Software, San Diego, California, USA), and *p* values < 0.05 were considered as significant. The significance was denoted as * *p* < 0.05, ** *p* < 0.01.

## Data Availability

Raw data is available on request from the corresponding author (E.B.)

## Supplementary Materials

The following are available online at https://www.mdpi.com/article/10.3390/ijms221910784/s1.
