# Supplementary material for: GPR21 Inhibition Increases Glucose-Uptake in HepG2 Cells
Source: Int J Mol Sci. 2021 Oct 5;22(19):10784. doi: 10.3390/ijms221910784 (PMC8509304; doi:10.3390/ijms221910784)
Supplement: Supplementary file 1 [file ijms-22-10784-s001.zip › Figure 1Sproof.pptx]

## Slide 1
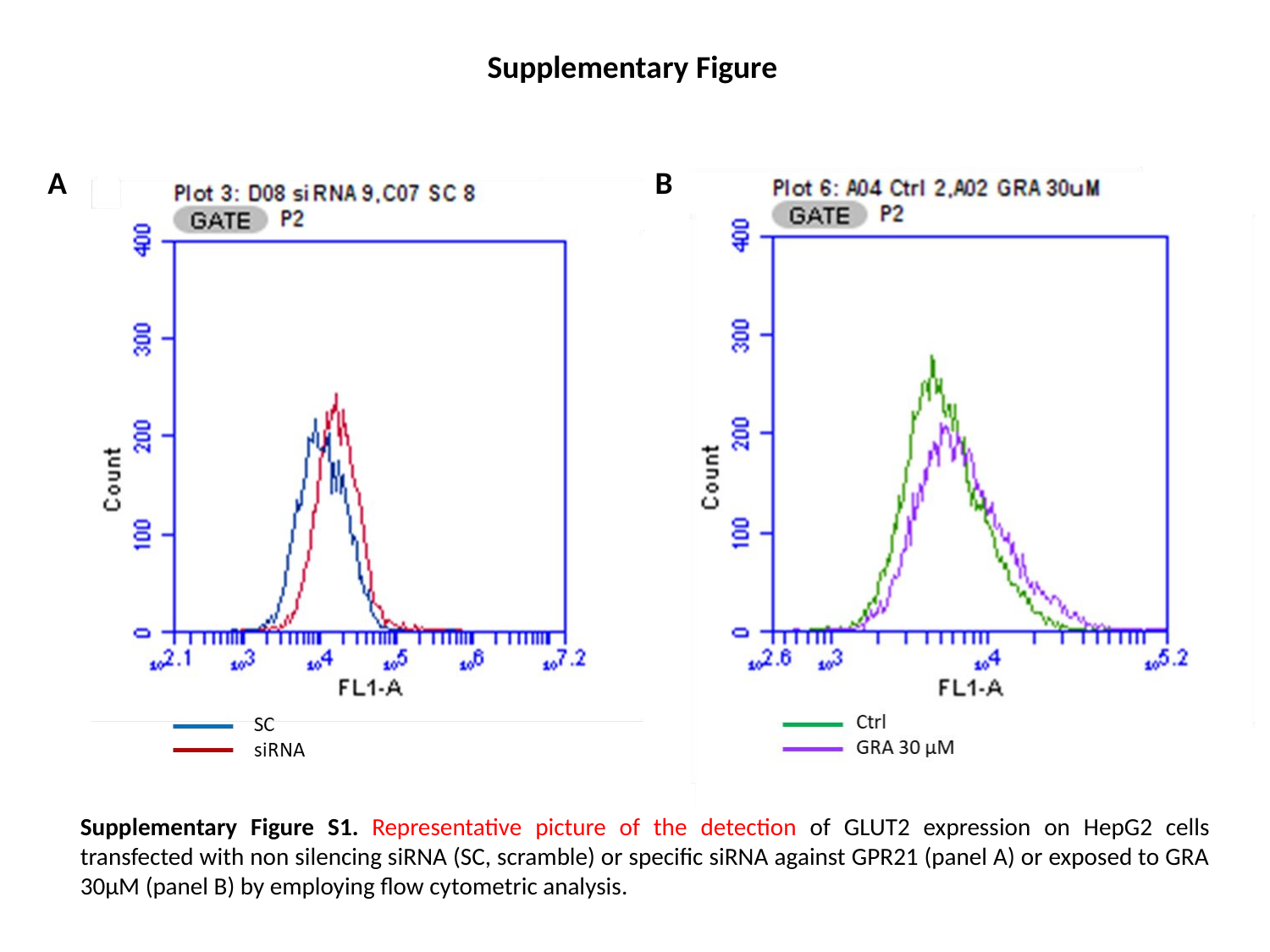

Supplementary Figure
A
B
Supplementary Figure S1. Representative picture of the detection of GLUT2 expression on HepG2 cells transfected with non silencing siRNA (SC, scramble) or specific siRNA against GPR21 (panel A) or exposed to GRA 30μM (panel B) by employing flow cytometric analysis.
